# Seeds of *Cucurbita maxima* and *Carica papaya* are effective for controlling monogeneans in the gills of *Leporinus macrocephalus*

**DOI:** 10.1590/S1984-29612022029

**Published:** 2022-06-06

**Authors:** Luciano Pereira Negreiros, Vitória Moura Carvalho, Tiago Araújo Lima, Eliane Xavier Sousa, Marcos Tavares-Dias

**Affiliations:** 1 Instituto Federal do Acre – IFAC, Rio Branco, AC, Brasil; 2 Embrapa Amapá, Macapá, AP, Brasil

**Keywords:** Monogenea, phytotherapy, parasite, treatment, Monogenea, fitoterapia, parasito, tratamento

## Abstract

This study was carried out to evaluate the anthelminthic efficacy of seeds of *Cucurbita maxima* and *Carica papaya* for controlling monogeneans in the gills of *Leporinus macrocephalus*, besides hepatosomatic and splenosomatic index and condition factor of host. The fish were fed with seeds of *C. maxima* or *C. papaya* for seven days, and these treatments did not cause any mortality among them. *Jainus leporini*, *Urocleidoides paradoxus*, *Urocleidoides eremitus* and *Tereancistrum parvus* were the monogeneans found, and their prevalence in fish fed with seeds of *C. papaya* was 100%, while in fish fed with *C. maxima* the prevalence was 42.8%. Fish fed with seeds of *C. papaya* showed decreased in intensity and abundance of monogeneans, while fish fed with seeds of *C. maxima* presented decreased in abundance. Feeding of *L. macrocephalus* with seeds of *C. maxima* or *C. papaya* had efficacy of 69.6 and 67.8%, respectively. The hepatosomatic index of fish fed with seeds of *C. maxima* or *C. papaya* was not affected by the treatments. However, the splenosomatic index and condition factor of fish fed with *C. maxima* seeds decreased. Seeds of *C. maxima* and *C. papaya* may be used for controlling monogeneans of *L. macrocephalus* in fish farming.

## Resumo

Este estudo avaliou a eficácia antihelmíntica de sementes de *Cucurbita maxima* e *Carica papaya* no controle de monogenéticos das brânquias de *Leporinus macrocephalus*, além do índice hepatossomático e esplenossomático e do fator de condição do hospedeiro. Os peixes foram alimentados com sementes de *C. maxima* ou *C. papaya* durante sete dias, e os tratamentos não causaram mortalidade. Monogenéticos *Jainus leporini*, *Urocleidoides paradoxus*, *Urocleidoides eremitus* e *Tereancistrum parvus* tiveram prevalência de 100% nos peixes alimentados com sementes de *C. papaya*, enquanto nos peixes alimentados com *C. maxima* a prevalência foi de 42,8%. Os peixes alimentados com sementes de *C. papaya* apresentaram diminuição na intensidade média e abundância média de monogenéticos, enquanto os peixes alimentados com sementes de *C. maxima* apresentaram uma diminuição na abundância média. A alimentação de *L. macrocephalus* com sementes de *C. maxima* ou *C. papaya* teve eficácia de 69,6 e 67,8%, respectivamente. O índice hepatossomático dos peixes alimentados com sementes de *C. maxima* ou *C. papaya* não foi afetada pelos tratamentos. No entanto, o índice esplenossomático e o fator de condição dos peixes alimentados com sementes de *C. maxima* diminuíram. Sementes de *C. maxima* e *C. papaya* podem ser usadas no controle de monogenéticos das brânquias de *L. macrocephalus* em piscicultura.

## Introduction

Fish are an important source of protein because of their short production cycle. Fish aquaculture plays an important socioeconomic role around world, including in Brazil. Around one million people are directly employed in fish farming in Brazil, and this generates another two million indirect jobs. In 2021, Brazilian fish farming generated US$ 1.5 billion in revenues (PeixeBR, 2022). However, intensification of fish aquaculture has led to increased levels of diseases due to problems of inadequate management. Infection by Monogenea is one of the diseases thus caused. Controlling parasitic diseases within the fish aquaculture industry helps to raise its economic and socioeconomic levels.

Monogeneans are parasites in the phylum Platyhelminthes that have a short and direct life cycle. Their vertical transmission facilitates infection levels within fish aquaculture (Alves et al., 2019; Cruz et al., 2022). Their pathogenicity is directly associated with organ fixation, infection intensity, feeding strategy and general host health (Cruz et al., 2022). Hence, it is difficult to control these ectoparasites in fish farming.

Many chemical drugs have been used against these parasites, but they all have some drawbacks, e.g., low efficacy, toxicity to hosts and environmental and human health problems (Alves et al., 2019; Cruz et al., 2022). In addition, long-term use of chemical drugs can lead to drug resistance among these parasites (Jeyavani et al., 2022). Hence, increasing attention has been paid to the use of traditional plant-based medicines and their bioactive products (phytotherapy) for controlling parasitic diseases in fish aquaculture (Fujimoto et al., 2012; Tavares-Dias, 2018; Trasviña-Moreno et al., 2019; Zhu, 2020; Jeyavani et al., 2022). Herbal therapy does not significantly pollute the aquaculture environment, and is not toxic to humans, thereby ensuring food safety (Zhu, 2020).

Among the herbal products used as alternative methods for controlling the diseases caused by helminths in different animal species are the seeds of pumpkins (*Cucurbita* spp.) (Feitosa et al., 2013; Grzybek et al., 2016; Marie-Magdeleine et al., 2009; Lima et al., 2020) and papaya (*Carica papaya*) (Shaziya & Goyal, 2012; Fujimoto et al., 2012; Feroza et al., 2017; Trasviña-Moreno et al., 2019). Both of these phytotherapeutics are relatively inexpensive alternatives, compared with the currently available chemotherapeutics, and have been considered to be good candidates for providing anthelminthic control.

As there is an urgent need for innovative methods that could act towards controlling the diseases caused by monogeneans in farmed fish, use of both *Cucurbita maxima* (Fujimoto et al., 2012) and *Carica papaya* (Fujimoto et al., 2012; Trasviña-Moreno et al., 2019) has been recommended. However, these phytotherapeutics have not been used for control and treatment against monogeneans in *Leporinus macrocephalus*, a fish that is reared in Brazilian aquaculture. Thus, the aim of the present study was to investigate the anthelminthic efficacy of *C. maxima* and *C. papaya* seeds for controlling monogeneans in the gills of *L. macrocephalus*, as well as hepatosomatic index, splenosomatic index and condition factor of host.

## Materials and Methods

### Fish and monogenean parasites

One hundred fingerlings of *L. macrocephalus* were obtained from a commercial fish farm in Rio Branco, in the state of Acre, Brazil, and were kept at the laboratory of the Instituto Federal do Acre (IFAC), Campus Baixada do Sol, in Rio Branco, Brazil. The fish were acclimatized for 10 days in a 1,000 L tank, with constant water flow and aeration, and were fed twice a day with commercial feed containing 35% crude protein (Guabi, Brazil). The following water parameters were maintained in the tanks: temperature at 29.1 ± 0.1 °C, dissolved oxygen at 5.6 ± 0.2 mg/L, pH at 5.4 ± 0.2, total ammonia at 0.4 ± 0.01 mg/L, alkalinity at 12.0 ± 0.1 mg/L and water hardness at 12.0 ± 0.1 mg/L. The fish excrement and feed residues accumulated in the bottom of the tanks was removed once every day. This stock of fish was used in all the *in vivo* assays.

The monogeneans used in these experiments were obtained from naturally infested fish.

### Obtaining and preparing of *Cucurbita maxima* and *Carica papaya* seeds

*Cucurbita maxima* and *C. papaya* were acquired from commercial sources in the city of Rio Branco, state of Acre, Brazil. The seeds of *C. maxima* and *C. papaya* were removed and dried in an oven at 50 °C for 24 h. Subsequently, these seeds were crushed using a meat grinder to produce particles of sizes around 6 mm.

### Feeding with seeds of *Cucurbita maxima* and *Carica papaya*

Fifty-four fingerlings of *L. macrocephalus* (6.9 ± 1.4 cm and 4.6 ± 3.8 g) that were naturally parasitized by monogeneans were randomly distributed into nine 250 L tanks. They were kept in a static water system under constant aeration for seven days. The following water parameters were maintained in the tanks: temperature at 30.1 ± 0.1 °C, dissolved oxygen at 5.5 ± 0.2 m /L, pH at 5.6 ± 0.2, total ammonia at 0.4 ± 0.01 mg/L, alkalinity at 11.0 ± 0.1 mg/L and water hardness at 11.0 ± 0.1 mg/L. The fish excrement and feed residues accumulated in the bottom of the tanks was removed once every day.

Control fish were fed once a day *ad libitum* with commercial feed containing 35% crude protein (Guabi, Brazil), for seven days. This control group consisted of three replicates with six fish each (18 fish per treatment). One group with three replicates of six fish each (18 fish per treatment) was fed once a day *ad libitum* with crushed seeds of *C. maxima*, for seven days. Another group with three replicates of six fish each (18 fish per treatment) was fed once a day *ad libitum* with crushed seeds of *C. papaya*, for seven days. The leftovers of crushed seeds were removed from the tanks every day.

After seven days of feeding with crushed seeds of *C. maxima* and *C. papaya*, the fish were euthanized by means of medullary section, weighed (g) and measured (cm). Their gills were excised, fixed in 5% formalin and examined under a stereomicroscope to identify and quantify the monogenean parasites. The parasites were prepared for identification as recommended by Eiras et al. (2006). After parasite quantification, the prevalence and mean intensity of infection were calculated as described by Bush et al. (1997) and the efficacy of each treatment as described by Fujimoto et al. (2012). Liver and spleen weight were measured and were used to determine the splenosomatic index (SSI) and hepatosomatic index (HSI) of fish (Tavares-Dias et al., 2000). Body weight and length were used to determine the relative condition factor (Kn) (Le Cren, 1951).

The data were evaluated based on the Shapiro–Wilk normality test and Bartlett’s test of homoscedasticity. Because the intensity and abundance data were not normally distributed, they were analyzed by the Kruskal–Wallis test, followed by Dunn’s test for comparison among medians (Zar, 2010).

This study was developed in accordance with the principles adopted by the Brazilian College of Animal Experimentation (COBEA) and with authorization from the Ethics Committee in the Use of Animals of Embrapa Amapá (Protocol No. 013-CEUA/CPAFAP).

## Results and Discussion

The hepatosomatic index, length and weight of *L. macrocephalus* fed with seeds of *C. maxima* or *C. papaya* for seven days were not affected by the treatments. However, the splenosomatic index and condition factor of *L. macrocephalus* fed with *C. maxima* seeds decreased (Table 1). The hepatosomatic index is an indirect measurement of glycogen and carbohydrate levels, and can be used to indicate the nutritional state of the fish. The splenosomatic index is a measurement of both the immune status and the hematopoietic capacity of the fish (Tavares-Dias et al., 2000; Voorhees et al., 2019). Therefore, the results from *L. macrocephalus* indicated that the growth and body condition was negatively influenced probably by the reduction in ingestion of food, and also by the immune status and hematopoiesis.

**Table 1:** Infection rates by monogeneans and body parameters of *Leporinus macrocephalus* fed with seeds of *Cucurbita maxima* and *Carica papaya* during seven days.

**Parameters**	**Control**	***Carica papaya* seed**	***Cucurbita maxima* seed**
Length (cm)	6.3 ± 1.0a	7.6 ± 1.4a	7.2 ± 1.6a
Weight (g)	3.1 ± 2.5b	6.3 ± 4.3b	5.1 ± 4.9b
Prevalence (%)	100	100	42.8
Mean intensity	12.1 ± 14.3a	3.3 ± 2.1b	8.2 ± 12.6ab
Mean abundance	12.1 ± 14.3a	3.3 ± 2.1b	3.9 ± 9.2b
HSI (%)	1.3 ± 0.3a	1.3 ± 0.3a	1.1 ± 0.4a
SSI (%)	0.9 ± 1.1a	0.7 ± 0.7a	0.3 ± 0.2b
Kn	0.90 ± 1.16a	1.01 ± 0.31a	0.69 ± 0.40b

Values express mean ± Standard deviation. Different letters in same line indicate differences by the Dunn test (p>0.05). SSI: Splenosomatic index, HIS: Hepatosomatic index, Kn: Relative condition factor.

In the present study, none of the treatments with diets containing seeds of *C. maxima* or *C. papaya* caused any mortality of *L*. *macrocephalus* over the seven-day period. The *Leporinus macrocephalus* specimens were naturally infected by *Jainus leporini*, *Urocleidoides paradoxus*, *Urocleidoides eremitus* and *Tereancistrum parvus*, which showed prevalence of 100% among the fish fed with seeds of *C. papaya* for seven days, while among the fish fed with *C. maxima* the prevalence was lower. In addition, the fish fed with seeds of *C. papaya* showed lower mean intensity and mean abundance of monogeneans, while the fish fed with seeds of *C. maxima* presented lower mean abundance of monogeneans (Table 1).

**Figure 1:**
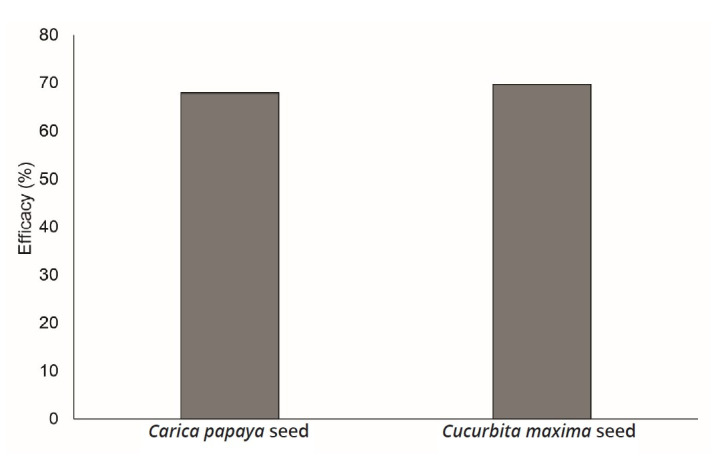
Antiparasitic efficacy of *Cucurbita maxima* and *Carica papaya* seeds against monogeneans of gills of *Leporinus macrocephalus*.

Disease management and control are difficult, particularly because of the limited availability of low-cost licensed drugs with proven efficacy (Alves et al., 2019). Furthermore, drugs need to be safe and effective, and therapeutic agents must also present low toxicity when used to treated fish. Feeding of *L. macrocephalus* with seeds of *C. maxima* or *C. papaya*, for seven days, showed efficacy of 69.6 and 67.8%, respectively (Figure 1). Similar results were previously reported with regard to the efficacy of treatments against monogeneans in *Astyanax zonatus* (72%), after these fish were fed with seeds of *C. papaya* for seven days. This contrasted with other results from *C. maxima*, in which the efficacy was shown to only be 39% (Fujimoto et al., 2012). Water-ethanol extracts of *C. papaya* did not show antiparasitic properties against *Neobenedenia* sp. in *Seriola lalandi*, probably because of instability or low concentration of the bioactive compounds used (Trasviña-Moreno et al., 2019).

The anthelmintic activity of *C. papaya* seeds has been attributed to the compound benzyl isothiocyanate, along with presence of carpaine and carpasemine (Kermanshai et al., 2001; Krishna et al., 2008; Trasviña-Moreno et al., 2019). It is believed that the anthelmintic activity of the seeds of *Cucurbita* spp. is due to cucurbitacin B, cucurbitin (3-amino-pyrrolidine-3-carboxylic acid), saponins and sterols, but roles for other compounds such as cucurmosin, berberine and palmatine possibly cannot be ruled out (Marie-Magdeleine et al., 2009; Grzybek et al., 2016).

In conclusion, our results demonstrated that *C. maxima* and *C. papaya* seeds were effective for controlling monogeneans in the gills of *L. macrocephalus*. Therefore, their use may be signaled as an alternative anthelminthic for controlling and treating monogenean infections in this fish in fish farming.
